# Development of Self-Associating SN-38-Conjugated Poly(ethylene oxide)-Poly(ester) Micelles for Colorectal Cancer Therapy

**DOI:** 10.3390/pharmaceutics12111033

**Published:** 2020-10-29

**Authors:** Sams M. A. Sadat, Mohammad Reza Vakili, Igor M. Paiva, Michael Weinfeld, Afsaneh Lavasanifar

**Affiliations:** 1 Faculty of Pharmacy and Pharmaceutical Sciences, University of Alberta, Edmonton, AB T6G 2E1, Canada; sadat@ualberta.ca (S.M.A.S.); paiva@ualberta.ca (I.M.P.); 2 Department of Oncology, Faculty of Medicine and Dentistry, University of Alberta, Edmonton, AB T6G 2R7, Canada; michael.weinfeld@albertahealthservices.ca; 3 Department of Experimental Oncology, Cross Cancer Institute, Edmonton, AB T6G 1Z2, Canada; 4 Department of Chemical and Material Engineering, University of Alberta, Edmonton, AB T6G 1H9, Canada

**Keywords:** SN-38, irinotecan, chemotherapy, polymeric micelle, colorectal cancer, mPEO-*b*-PBCL, mPEO-*b*-PCCL

## Abstract

The clinical use of 7-ethyl-10-hydroxy-camptothecin (SN-38), which is the active metabolite of irinotecan, has been hampered because of its practical water-insolubility. In this study, we successfully synthesized two self-associating SN-38-polymer drug conjugates to improve the water-solubility of SN-38, while retaining its anticancer activity. The polymeric micellar SN-38 conjugates were composed of either methoxy-poly(ethylene oxide)-block-poly(α-benzyl carboxylate-ε-caprolactone) conjugated to SN-38 at the PBCL end (mPEO-*b*-PBCL/SN-38) or mPEO-block-poly(α-carboxyl-ε-caprolactone) attached to SN-38 from the pendent-free carboxyl site (mPEO-*b*-PCCL/SN-38). The chemical structure of block copolymers was confirmed by ^1^H NMR. The physicochemical characterizations of their self-assembled structures including size, surface charge, polydispersity, critical micellar concentration, conjugation content and efficiency, morphology, kinetic stability, and in vitro release of SN-38 were compared between the two formulations. In vitro anticancer activities were evaluated by measuring cellular cytotoxicity and caspase activation by MTS and Caspase-Glo 3/7 assays, respectively. The hemolytic activity of both micellar structures against rat red blood cells was also measured. The results showed the formation of SN-38-polymeric micellar conjugates at diameters < 50 nm with a narrow size distribution and sustained release of SN-38 for both structures. The loading content of SN-38 in mPEO-*b*-PBCL and mPEO-*b*-PCCL were 11.47 ± 0.10 and 12.03 ± 0.17 (% *w/w*), respectively. The mPEO-*b*-PBCL/SN-38, end-capped micelles were kinetically more stable than mPEO-*b*-PCCL/SN-38. The self-assembled mPEO-*b*-PBCL/SN-38 and mPEO-*b*-PCCL/SN-38 micelles resulted in significantly higher cytotoxic effects than irinotecan against human colorectal cancer cell lines HCT116, HT-29, and SW20. The CRC cells were found to be 70-fold to 330-fold more sensitive to micellar SN-38 than irinotecan, on average. Both SN-38-incorporated micelles showed two-fold higher caspase-3/7 activation levels than irinotecan. The mPEO-*b*-PBCL/SN-38 micelles were not hemolytic, but mPEO-*b*-PCCL/SN-38 showed some hemolysis. The overall results from this study uphold mPEO-*b*-PBCL/SN-38 over mPEO-*b*-PCCL/SN-38 micellar formulation as an effective delivery system of SN-38 that warrants further preclinical investigation.

## 1. Introduction

Cancer-related mortality has increased by ~40% over the past 40 years and is anticipated to show a further 60% increase by 2030 [1]. Among all cancerous diseases, colorectal cancer (CRC) is the second most fatal cancer worldwide. Approximately, 1.8 million new CRC cases occurred and 881,000 deaths were reported in 2018 [2]. For CRC patients with localized disease, surgery is a curative option. However, despite improved screening practices, CRC is commonly diagnosed at advanced stages where metastasis to nearby or distant organs is seen. In CRC patients with metastatic disease, the curative benefit of first-line surgical interventions is limited. Patients with de novo metastatic disease or those showing relapse and advancement to metastatic stage after the first round of therapy make up more than 60% of CRC patients [3]. Chemotherapy and radiotherapy are the leading treatment strategies in these patients and are usually used either to control unresectable tumor growth and its further spread or reduce the size of locally metastasized cancer, making the patient a candidate for surgery and tumor removal at the metastatic site [4].

Irinotecan is a water soluble prodrug of SN-38 and a DNA topoisomerase I (Topo-I) inhibitor approved by the FDA for treating CRC [5]. After enzymatic activation in liver and cancer cells, <10% of irinotecan are converted to a biologically active metabolite; i.e., 7-ethyl-10-hydroxy-camptothecin (SN-38), which is 100–1000 times more potent than irinotecan [6]. To achieve the therapeutic effect, a much higher dose of irinotecan is required. Moreover, the conversion of irinotecan to SN-38 depends on the genetic polymorphism or inter-individual variability of carboxylesterase activity [7,8]. The administration of high irinotecan doses in CRC patients is also associated with observavtion of severe side effects such as diarrhea, myelosuppression, acute cholinergic-like syndrome, and neutropenia [9].

The direct clinical use of SN-38 exhibits an appealing alternative to avoid the dose-limiting side-effects of irinotecan and overcome its less than optimal potency. However, the clinical use of SN-38 is limited by its inherent poor water-solubility and biological inactivation through the glucuronidation pathway [10]. To solve these problems, a number of approaches have been explored, among which, the nanoparticle-based delivery of SN-38 has gained the most attention due to the additional advantage in passive targeting of solid tumors by nano-carriers [11,12,13,14,15]. Among different nano-carriers tried for delivery of SN-38, supramolecular polymeric micellar structures stand out [16]. This is due to the potential of polymeric micellar structures to solubilize SN-38 and protect it within their hydrophobic core from the destabilizing effect of the biological milieu, leading to its enhanced biological stability while preserving SN-38 toxicity against cancer cells [12].

**Figure 1:**
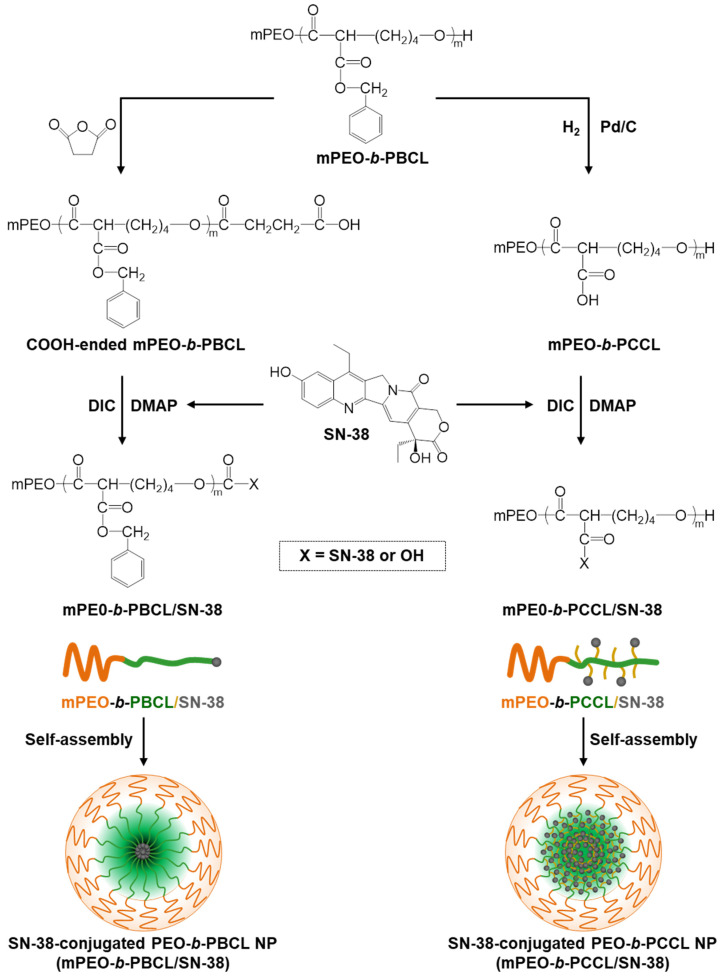
Chemical structures of SN-38, mPEO-*b*-PBCL, mPEO-*b*-PCCL, and schematic procedures to synthesize mPEO-*b*-PBCL/SN-38 and mPEO-*b*-PCCL/SN-38, forming self-assembled micelles.

The main objective of the present work was to develop a polymeric micellar formulation of SN-38 in biodegradable nanocarriers based on poly(ethylene oxide)-poly(ester) block copolymers. For this purpose, conjugation of SN-38 to pendant carboxyl functional groups on methoxy-poly(ethylene oxide)-block-poly(α-carboxyl-ε-caprolactone) (mPEO-*b*-PCCL) or end-capped functional groups on methoxy-poly(ethylene oxide)-block-poly(α-benzyl carboxylate-ε-caprolactone) (mPEO-*b*-PBCL) was pursued (Figure 1). This strategy was expected to enhance the solubilized levels of SN-38 in aqueous media. The results of comparative studies on physicochemical properties, kinetic and thermodynamic stability, in vitro cytotoxicity, and hemolytic activity of the two generated polymeric micellar formulations, i.e., mPEO-*b*-PCCL/SN-38 and mPEO-*b*-PBCL/SN-38, are presented here.

Similar strategies have been reported before by the conjugation of SN-38 through its free hydroxyl group to other carriers [17,18,19,20]. An SN-38 conjugated to micelle-forming PEO-poly(glutamic acid) formulation, known as NK012, has completed phase II clinical trials in triple-negative breast cancer and relapsed small cell lung cancer [21,22]. The SN-38 polymeric micellar drug conjugates that are the subject of current study may prove to be beneficial when compared to NK012 due to the more hydrophobic nature of their core-forming block compared to poly(glutamic acid), leading to improved stability. The proven biodegradability of the poly(ester) core may be considered an additional potential advantage over poly(amino acid) structures.

## 2. Materials and Methods

### 2.1. Materials

Methoxy-polyethylene oxide (mPEO) (average molecular weight of 5000 g mol^−1^), sodium dodecyl sulfate (SDS), palladium on charcoal, and bovine serum albumin (BSA) and all research grade organic solvents were purchased from Sigma (St. Louis, MO, USA). α-Benzyl carboxylate-ε-caprolactone monomer was synthesized by Alberta Research Chemicals Inc. (Edmonton, AB, Canada). Stannous octoate was purchased from Sigma-Aldrich, purified, and dehydrated by toluene azeotropic distillation, which is followed by vacuum distillation. (S)-4, 11-Diethyl-4, 9-di-OH-1, 12-dihydro-4*H*-2-oxa-6,12a-diaza-dibenzo[b,h]fluorene-3,13-dione (SN-38) (purity > 97%) was purchased from abcr GmbH (Karlsruhe, Germany). All other chemicals and reagents used were of an analytical grade.

### 2.2. Synthesis of Block Copolymers

The copolymers of mPEO-*b*-PBCL with two different degrees of polymerization (DP = 12 and DP = 20) were synthesized by ring-opening polymerization of α-benzyl carboxylate-ε-caprolactone using mPEO (MW: 5000 g mol^−1^) as an initiator and stannous octoate as a catalyst, according to a previously described method [23]. In the first step, 2 g and 0.5 g of mPEO were added to 1.19 g and 0.6 g of α-benzyl carboxylate-ε-caprolactone (BCL) monomer to prepare two products with DPs of 12 and 20, respectively. Stannous octoate (0.1% *w/w* of the polymer) was then added to an ampoule and sealed under vacuum. The sealed ampoule was kept in an oven for 4 h at 140 °C. After 4 h of a polymerization reaction, the ampoule was cooled down to room temperature to stop the polymerization reaction. In the second step, block copolymers were dissolved in dichloromethane and, subsequently, precipitated in hexane and centrifuged at 3000 rpm to discard the supernatant. The final product was washed twice with hexane and completely dried in a vacuum oven overnight at room temperature.

The mPEO-*b*-PCCL copolymer was synthesized by catalytic debenzylation of mPEO-*b*-PBCL in the presence of H_2_, according to a method described previously [24]. A solution of mPEO-*b*-PBCL (1 g) in 50 mL anhydrous tetrahydrofuran (THF) was prepared and placed into a cylindrical flask where palladium on charcoal (300 mg) was dispersed. The mixture was vigorously stirred with a magnetic stirrer under continuous H_2_ gas flow at 0.2 Lmin^−1^ for 8 h at room temperature. Afterward, the mixture was centrifuged at 3000 rpm to remove the catalyst. The collected supernatant was evaporated to precipitate the product. mPEO-*b*-PCCL was washed with diethyl ether repeatedly to remove impurities. The final product was dried under vacuum for 48 h at room temperature.

### 2.3. Synthesis of Carboxyl-Terminated mPEO-b-PBCL Block Copolymers

The mPEO-*b*-PBCL copolymer was chemically modified by a reaction with succinic anhydride to generate mPEO-*b*-PBCL copolymer, the end-capped carboxylic acid functional group (mPEO-*b*-PBCL-COOH). First, 1 g of mPEO-*b*-PBCL and 1.5 times molar excess of succinic anhydride were mixed and placed in a previously flame-dried ampoule. The ampoule was sealed and kept in an oven for 4 h reaction at 140 °C. Thereafter, COOH-terminated block copolymers were dissolved in dichloromethane and, subsequently, precipitated in hexane and centrifuged at 3000 rpm to discard the supernatant [25]. The final product was washed twice with hexane and completely dried in a vacuum oven overnight at room temperature.

### 2.4. Conjugation of SN-38 to mPEO-b-PBCL-COOH Copolymers

Conjugation of SN-38 to mPEO-*b*-PBCL was conducted by activation of the carboxylic acid terminal group on the PBCL block using *N*,*N*′-diisopropylcarbodiimide (DIC) and 4-dimethylaminopyridine (DMAP). At first, 21 mg of SN-38 and 13 mg of DMAP were dissolved together in 2 mL of anhydrous dimethylformamide (DMF). Separately, 0.2 g (0.026 mmoles) of mPEO-*b*-PBCL-COOH copolymer (DP = 12 for PBCL block) was dissolved in 2 mL of DMF. The vial containing the polymer solution was placed in an ice-water bath and stirred under Ar gas. DIC (100 μL) was added to the solution at 0 °C and kept for 20 min. The solution of SN-38 and DMAP was then added to the reaction solution. The reaction solution was stirred under Ar gas for 48 h at room temperature. The reaction mixture was then diluted with 6 mL of dimethyl sulfoxide (DMSO) and dialyzed against DMSO for 48 h and deionized water overnight to remove unreacted SN-38 and other impurities and by-products. The solution was freeze-dried to obtain a dry product.

### 2.5. Conjugation of SN-38 to mPEO-b-PCCL Copolymers

Conjugation of SN-38 to mPEO-*b*-PCCL was conducted by activation of carboxylic acid pendant groups on the PCCL block using *N*,*N*′-diisopropylcarbodiimide (DIC) and 4-dimethylaminopyridine (DMAP). At first, 50 mg of SN-38 and 39 mg of DMAP were dissolved together in 4 mL of DMF. Separately, 0.25 g (0.031 mmoles) of the mPEO-*b*-PCCL copolymer (DP = 20, for the second bock) was dissolved in 3 mL of DMF. The vial containing the polymer solution was placed in an ice-water bath. The solution was then stirred under Ar gas. DIC (110 μL) was added at 0 °C to the polymer solution under Ar gas. The reaction container was kept for 20 min. The SN-38 and DMAP solution was then added to the reaction solution and were stirred under Ar gas for 48 h at room temperature. The reaction mixture was then diluted with 3 mL DMSO, dialyzed against DMSO for 48 h, and then deionized water overnight to remove unreacted SN-38 and other impurities and by-products. The final aqueous solution was freeze-dried to obtain the dry product.

### 2.6. Characterization of Block Copolymers and Drug-Copolymer Conjugates

The synthesized block copolymers and SN-38-copolymer conjugates were characterized for their number average molecular weights by ^1^H NMR (600 MHz Avance III-Bruker, East Milton, ON, Canada) using deuterated chloroform (CDCl_3_) as a solvent. The DP of the PBCL and PCCL blocks was calculated from the ^1^H NMR based on the ratio of the peak intensity of protons from the ethylene (-CH_2_CH_2_O-) moiety of PEO (δ = 3.65 ppm) to the peak intensity of the protons from the (-COOCH_2_-) methylene of caprolactone (δ = 4.05 ppm), considering a molecular weight of 5000 g mol^−1^ for the PEO block. The level of debenzylation of the mPEO-*b*-PCCL polymer was also measured based on ^1^H NMR. The yield of the hydrogenation reaction was determined by taking into account the signal of methylene protons from the benzyl group at ~7.4 ppm (-CH_2_-Ph) and the signal of methylene protons from the caprolactone backbone at 4.05 ppm (-O-CH_2_-C=O). The ratio of their integration was then multiplied by 100 in order to obtain the molar percentage of the remaining benzyl groups.

The SN-38 conjugation level was measured using a Synergy-H1 microplate reader (BioTEK Instruments Inc., Winooski, VT, USA) at a wavelength of 383 nm. The level of SN-38 conjugation was expressed as a loading percentage (% *w/w*) with respect to the carboxylic acid-terminated residue of mPEO-*b*-PBCL and α-carboxylic-ε-caprolactone residue of mPEO-*b*-PCCL.

### 2.7. Self-Assembly of Block Copolymers and Physicochemical Characterization of Self-Assembled Structures

The mPEO-*b*-PBCL, mPEO-*b*-PBCL/SN-38, mPEO-*b*-PCCL, and mPEO-*b*-PCCL/SN-38 micelles were prepared as previously described [26]. A total of 10 mg copolymers or drug-copolymer conjugates were completely dissolved in 1 mL of acetone. Then, the organic phase was transferred dropwise to 3 mL of an aqueous phase and left overnight under continuous stirring with a magnetic bar inside the fume hood to completely evaporate the organic solvent. The size (Z-average diameter), polydispersity index (PDI), zeta potential (ZP), and critical micellar concentration (CMC) of the micelles were measured by dynamic light scattering (DLS) using a Malvern Zetasizer 3000 (Malvern Instruments Ltd., Malvern, UK).

In order to investigate micellar stability, CMC of the formulations was determined by a DLS technique following a previously published method [24]. A series of micellar solutions of mPEO-*b*-PBCL, mPEO-*b*-PBCL/SN-38, mPEO-*b*-PCCL, and mPEO-*b*-PCCL/SN-38 with a concentration range of 1000 to 0.24 µg·mL^−1^ were prepared in glass vials. The intensity of the scattered light for prepared samples was detected at an angle of 173 °C under a single attenuator index. Measurements were carried out in polystyrene cells at 25 °C. The count rate (Kcps) as a function of the intensity of the scattered light was plotted against the concentration of copolymers and SN-38-copolymer conjugates.

To determine the stability of micelles against dissociation, kinetic stability was also measured by a DLS method as previously described [27]. In brief, micelles were prepared using individual copolymers with a concentration of 3 mg·mL^−1^ and incubated with an aqueous solution of a destabilizing agent, sodium dodecyl sulfate (SDS) (20 mg·mL^−1^) at a ratio of 2:1 (*v/v*). The intensity of scattered light and PDI were measured at different incubation time intervals (0, 1, 2, 4, 8, and 24 h).

### 2.8. Transmission Electron Microscopy (TEM)

The morphology of self-assembled structures under study was investigated by TEM using a Morgagni TEM (Field Emission Inc., Hillsboro, OR, USA) with a Gatan digital camera (Gatan, Pleasanton, CA, USA). Furthermore, 20 μL of the micellar solution with a polymer concentration of 1 mg·mL^−1^ was placed on a copper-coated grid. The grid was held horizontally for 1 min to allow the colloidal particles to settle down. The excess fluid was removed by filter paper. The copper-coated grids holding the aqueous samples were then negatively stained by 2% phosphotungstic acid. After 2 min, the excess fluid was removed by filter paper and the grid was loaded into TEM for image analysis.

### 2.9. In Vitro Drug Release

In vitro release of SN-38 from the self-assembled structures was investigated using a dialysis-bag diffusion technique. Each dialysis bag (Spectrapor dialysis tubing, MWCO = 3.5 kDa, Spectrum Laboratories, Rancho Dominguez, CA, USA.) containing 3 mL of the micellar formulation in water with a concentration of 3 mg·mL^−1^ or equivalent loaded concentration of free SN-38 dissolved in DMSO was immersed into 300 mL of release medium (4% albumin in ultrapure water to provide a sink for free drug release) maintained at 37 °C in a shaking water bath at 65 rpm (Julabo SW 22, Seelbach, Germany). At selected time intervals (0, 1, 2, 4, 6, 8, 24, 48, and 72 h), 300 μL of aliquots from inside of the dialysis bag were withdrawn and replaced with an equal volume of fresh release media (water). The concentrations of SN-38 in collected samples were determined by a UV–Vis spectrophotometer (BioTEK, Winooski, VT, USA). Detection was performed at a wavelength of 383 nm. All experiments were carried out in triplicate. Finally, the release profiles of the formulations were compared using the similarity factor, f_2_, and the profiles were considered significantly different if f_1_ ≤ 15 and f_2_ ≤ 50, according to the following equations.

(1) $$\mathrm{Difference}\;\mathrm{factor}\;(f_{1})=\left(\frac{\sum_{n}^{j=1}\left|\mathrm{Rj}-\mathrm{Tj}\right|}{\sum_{n}^{j=1}\mathrm{Rj}}\right)\times 100$$

(2) $$\mathrm{Similarity}\;\mathrm{factor}\;(f_{2})=50\;\log\;\left({\left[1+\left(\frac{1}{n}\right)\;\sum_{n}^{j=1}{\left|\mathrm{Rj}-\mathrm{Tj}\right|}^{2}\right]}^{-0.5}\times 100\right)$$

where n is the number of time points, Rj is the percent released of the reference at time point j, and Tj is the percent released of test formulations at time point j. 

### 2.10. Cell Lines

Three CRC cell lines, i.e., HCT116, HT-29, and SW620 C, were purchased from American Type Culture Collection (ATCC) (Manassas, VA, USA). The cells were cultured at 37 °C in 5% CO_2_ in a humidified incubator in a 1:1 mixture of Dulbecco’s modified Eagle medium and F12 (DMEM/F12) supplemented with 10% FBS, 50 U mL^−1^ penicillin, 50 mg·mL^−1^ streptomycin, 2 mmol L^−1^ l-glutamine, 0.1 mmol L^−1^ nonessential amino acids, and 1 mmol L^−1^ sodium pyruvate. All culture supplements were purchased from GIBCO Life Technologies Inc. (Burlington, ON, USA).

### 2.11. In Vitro Cytotoxicity Assay

The CellTiter 96^®^ AQueous One Solution Cell Proliferation Assay (MTS) kit was purchased from Promega, Madison, WI, USA and used to assay the cytotoxicity of SN-38, irinotecan, mPEO-*b*-PBCL, mPEO-*b*-PCCL, mPEO-*b*-PBCL/SN-38, and mPEO-*b*-PCCL/SN-38 against HCT116, HT-29, and SW620 cells, according to the manufacturer’s protocol. In brief, 2 × 10^3^ cells were plated in each well of 96-well flat-bottomed plates 24 h prior to the treatments. Then, cells were treated with the formulations at the concentration ranges of 0.001 to 100 µM for SN-38 and 0.00334 to 334 μg mL^−1^ for polymeric micellar SN-38 or empty polymeric micellar formulations. Control cells received only 0.1% DMSO. After different experimental incubation time points, 20 µL of the MTS reagent was added in each well and further incubated for 1 h at 37 °C before measuring the absorbance at 490 nm using a BioTEK microplate reader. The cell viability percentages were calculated using the following formula.

(3) $$\mathrm{Cell}\;\mathrm{viability}\;\left(\%\right)=\frac{\mathrm{Absorbance}\;\mathrm{of}\;\mathrm{treated}\;\mathrm{cells}-\mathrm{Absorbance}\;\mathrm{of}\;\mathrm{blank}\;\mathrm{well}}{\mathrm{Absorbance}\;\mathrm{of}\;\mathrm{untreated}\;\mathrm{cells}-\mathrm{Absorbance}\;\mathrm{of}\;\mathrm{blank}\;\mathrm{well}}\times 100$$

### 2.12. Caspase 3/7 Activity Measurements

The Caspase-Glo 3/7 assay reagent was purchased from Promega (Madison, WI, USA) and used according to the manufacturer’s protocol to detect and quantify the in vitro caspase activity of free SN-38, irinotecan, mPEO-*b*-PBCL, mPEO-*b*-PCCL, mPEO-*b*-PBCL/SN-38, and mPEO-*b*-PCCL/SN-38 in HCT116, HT-29, and SW620 cell lines. The cells were first seeded onto 96-well plates at a density of 2 × 10^3^ cells per well and incubated for 24 h. Cells were then treated with the media containing various formulations at a final concentration equivalent to the respective IC_50_ concentrations of free SN-38, irinotecan, mPEO-*b*-PBCL/SN-38, and mPEO-*b*-PCCL/SN-38 for 6 h. The concentrations of mPEO-*b*-PBCL and mPEO-*b*-PCCL in each well were equivalent to the concentrations of these polymers in mPEO-*b*-PBCL/SN-38 and mPEO-*b*-PCCL/SN-38 treatments, respectively. Control cells received only 0.1% DMSO. After 6 h of incubation, the Caspase-Glo 3/7 assay reagent was added in each well and kept at room temperature for 45 min. The relative luminescence was measured and analyzed when compared to the control. Each experiment was carried out in triplicate.

### 2.13. Hemolytic Activity Assessment

Whole blood was collected from 22 weeks old Sprague-Dawley rats (~300 g) in heparinized tubes (BD Vacutainer, Toronto, ON, Canada) by cardiac puncture under anesthesia. Erythrocytes were separated from the heparinized blood by centrifugation at 2000× *g* for 10 min and then washed twice with isotonic PBS (pH 7.4). Separated erythrocytes were re-suspended and diluted in PBS to a final cell count of 2 × 10^7^ cells per mL. The aliquots of 200 µL cell suspension were treated with various concentrations of mPEO-*b*-PBCL, mPEO-*b*-PCCL, mPEO-*b*-PBCL/SN-38, and mPEO-*b*-PCCL/SN-38 and incubated at 37 °C with gentle mixing for 30 min. After the incubation, the tubes were centrifuged at 2000× *g* for 15 min to remove the unlysed erythrocytes and the supernatants containing released hemoglobin were transferred into 96-well plates. The absorbance of hemoglobin was measured at a wavelength of 540 nm using a microplate reader. Isotonic PBS was used as a negative control and full hemolysis (positive control) was achieved by mixing the erythrocyte suspension with pure water. Finally, hemolytic activity was assayed as the percentage of hemolysis caused by various concentrations of the treatment groups when compared to hemolysis in pure water. The percentage of hemolyzed erythrocytes was calculated using the equation below.

(4) $$\%\;\mathrm{Hemolysis}=\frac{\left(\mathrm{Absorbance}\;\mathrm{of}\;\mathrm{sample}-\mathrm{Absorbance}\;\mathrm{of}\;\mathrm{negative}\;\mathrm{control}\right)\times 100}{\mathrm{Absorbance}\;\mathrm{of}\;\mathrm{positive}\;\mathrm{control}-\mathrm{Absorbance}\;\mathrm{of}\;\mathrm{negative}\;\mathrm{control}}$$

### 2.14. Statistical Analysis

Data are shown as mean ± standard deviation of at least three experiments. GraphPad Prism6software (GraphPad Software Inc., La Jolla, CA, USA) was used for statistical analysis. The significance of difference between groups was assessed using one-way ANOVA, which was followed by Tukey’s post-hoc analysis. If a significant difference was found among the groups, median ranks between pairs of groups were compared using the Mann-Whitney U test. Differences in physicochemical characterization of micellar formulations were also tested using the unpaired student’s *t*-test. A value of *p* ≤ 0.05 was considered as statistically significant in all experiments.

## 3. Results

### 3.1. Physicochemical Characterization

**Figure 2:**
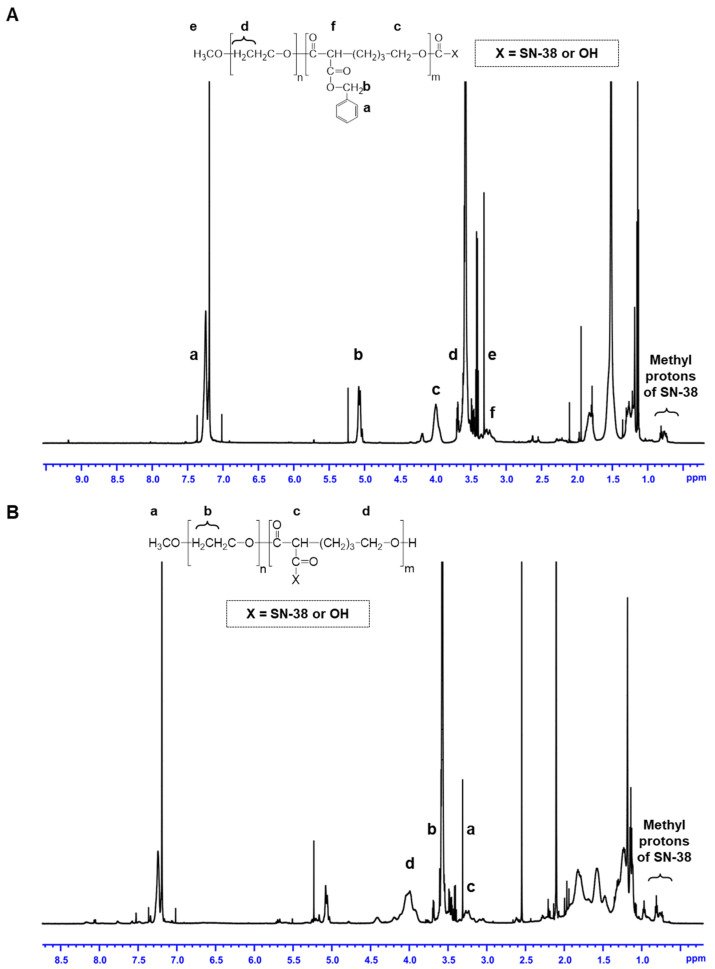
^1^H NMR spectra and corresponding peak assignments for (**A**) mPEO-*b*-PBCL/SN-38 and (**B**) mPEO-*b*-PCCL/SN-38.

The ^1^H NMR spectra for mPEO-*b*-PBCL/SN-38 and mPEO-*b*-PCCL/SN-38 and peak assignments are shown in Figure 2, while the ^1^H NMR spectra for free SN-38, mPEO-*b*-PBCL, and mPEO-*b*-PCCL are shown in Supplementary Figure S1, Figure S2, Figure S3 [24,28,29]. According to calculations based on the ^1^H NMR spectra, the DP was 12 and 20 for PBCL and PCCL blocks in MPEO-*b*-PBCL and mPEO-*b*-PCCL, respectively. The 100% catalytic hydrogenolysis conversion of mPEO-*b*-PBCL to mPEO-*b*-PCCL was also confirmed by ^1^H NMR in mPEO-*b*-PCCL. The conjugated content (% *w/w*) of SN-38 to mPEO-*b*-PBCL and mPEO-*b*-PCCL were 11.47 ± 0.10 and 12.03 ± 0.17, respectively, as measured by UV spectroscopy.

The physicochemical characteristics of the self-assembled mPEO-*b*-PBCL, mPEO-*b*-PCCL, mPEO-*b*-PBCL/SN-38, and mPEO-*b*-PCCL/SN-38 micelles including size, surface charge, PDI, and CMC are summarized in Table 1. The average diameters of micelles formed from mPEO-*b*-PBCL, mPEO-*b*-PCCL, mPEO-*b*-PBCL/SN-38, and mPEO-*b*-PCCL/SN-38 were below 100 nm and showed a relatively narrow polydispersity range below 0.2. As a result of the chemical conjugation of SN-38 to both copolymers, the average hydrodynamic diameters of mPEO-*b*-PBCL/SN-38 and mPEO-*b*-PCCL/SN-38 were significantly reduced (**** *p* < 0.0001) when compared to that of the SN-38-free block copolymers. The mean zeta potential of the micelles formed from mPEO-*b*-PBCL and mPEO-*b*-PCCL were neutral at a range from 0.04–0.09 mV. However, the mean zeta potential shifted toward negative when SN-38 was conjugated to mPEO-*b*-PBCL and mPEO-*b*-PCCL copolymers. Statistically, the presence of conjugated SN-38 in both block copolymer micelles resulted in significant changes in zeta potentials (mPEO-*b*-PBCL/SN-38, *p* = 0.01 and mPEO-*b*-PCCL/SN-38, *p* = 0.004 when compared to the micelles prepared using their respective SN-38-free block copolymers).

All self-assembled micelles showed CMC in the μg·mL^−1^ range as shown in Table 1. The measured CMCs of mPEO-*b*-PBCL, mPEO-*b*-PCCL, mPEO-*b*-PBCL/SN-38 and mPEO-*b*-PCCL/SN-38 were 4.43 ± 0.21, 3.88 ± 0.11, 69.92 ± 0.82, and 54.57 ± 0.12 µg·mL^−1^, respectively. After statistical analysis, the CMCs of mPEO-*b*-PCCL and mPEO-*b*-PCCL/SN-38 were significantly higher than that of the micelles prepared from mPEO-*b*-PBCL and mPEO-*b*-PBCL/SN-38. Notably, the CMC appeared to become lower upon conjugation of SN-38 irrespective of the polymer structure (mPEO-*b*-PBCL/SN-38, *p* = 0.03 and mPEO-*b*-PCCL/SN-38, *p* = 0.0008 when compared with their respective SN-38-free copolymeric micelles).

### 3.2. Transmission Electron Microscopy (TEM)

**Figure 3:**
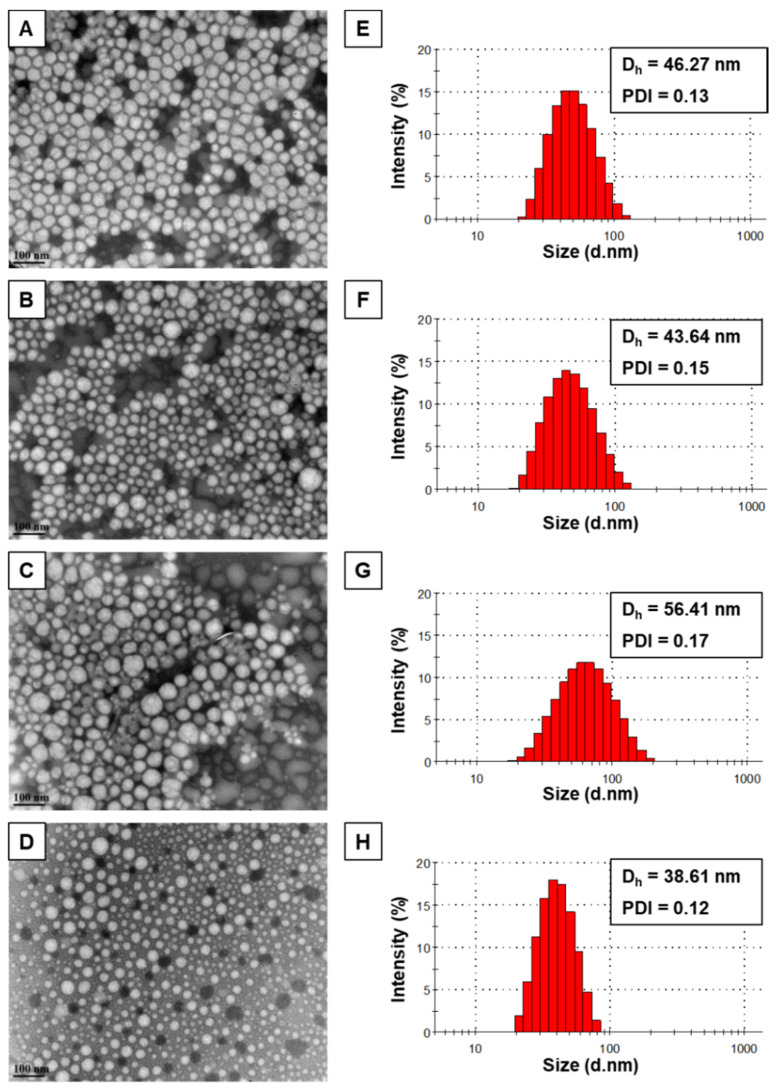
TEM images of the polymeric and SN-38-conjugated micelles formed from (**A**) mPEO-*b*-PBCL, (**B**) mPEO-*b*-PBCL/SN-38, (**C**) mPEO-*b*-PCCL, and (**D**) mPEO-*b*-PCCL/SN-38. Images were obtained at a magnification of 110,000× at 75 kV. The bar in the bottom left corner of each image indicates a scale of 100 nm. Hydrodynamic diameter (D_h_), polydispersity index (PDI), and size distribution of (**E**) mPEO-*b*-PBCL, (**F**) mPEO-*b*-PBCL/SN-38, (**G**) mPEO-*b*-PCCL, and (**H**) mPEO-*b*-PCCL/SN-38 micelles in aqueous medium were obtained using dynamic light scattering (DLS).

The morphology of the self-assembled structures was investigated by TEM images, confirming the formation of spherical-shaped mPEO-*b*-PBCL, mPEO-*b*-PCCL, mPEO-*b*-PBCL/SN-38, and mPEO-*b*-PCCL/SN-38 micelles with a uniform size (Figure 3). The size of the micelles was also measured from TEM images using ImageJ software. The average diameters of self-assembled mPEO-*b*-PBCL (47.8 ± 1.29 nm), mPEO-*b*-PCCL (55.82 ± 2.72 nm), mPEO-*b*-PBCL/SN-38 (44.98 ± 4.84 nm), and mPEO-*b*-PCCL/SN-38 (42.66 ± 3.58 nm) micelles obtained by DLS appeared to be similar. Moreover, a similar distribution pattern in the micellar population with a clear boundary was observed in TEM images of all micelles, indicating the lower aggregation tendency of micelles.

### 3.3. Kinetic Stability of Block Copolymeric Micelles

**Figure 4:**
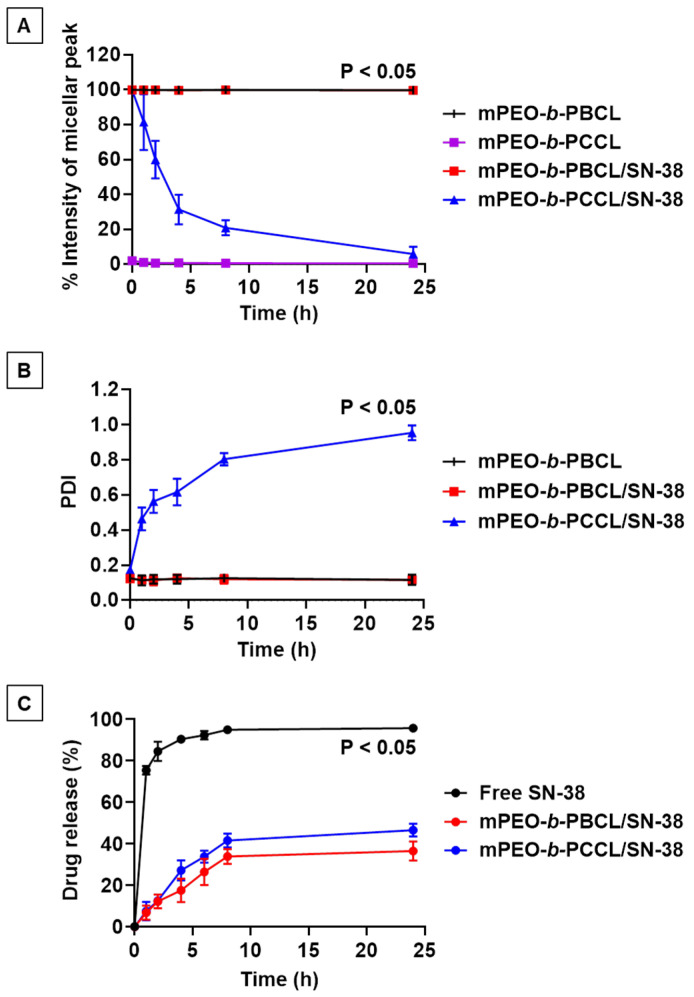
Average (**A**) percentage of intensity and (**B**) polydispersity index (PDI) of mPEO-*b*-PBCL, mPEO-*b*-PBCL/SN-38, mPEO-*b*-PCCL (no PDI data), and mPEO-*b*-PCCL/SN-38 micellar peak (3 mg·mL^−1^) in the presence of SDS (20 mg·mL^−1^) at a ratio of 2:1 (*v/v*) as a function of time up to 24 h. Each point represents mean ± SD (*n* = 3). (**C**) The drug release profile of mPEO-*b*-PBCL/SN-38 and mPEO-*b*-PCCL/SN-38 micelles compared to free SN-38 from dialysis tubing (MWCO = 3.5 kDa) in aqueous solution (4% albumin in ultrapure water) at 37 °C. Data are represented as mean ± SD (*n* = 3). The results of statistical analysis using one-way ANOVA followed by Tukey’s method showed a significant difference between PEO-*b*-PBCL and mPEO-*b*-PCCL, between mPEO-*b*-PBCL/SN-38 and mPEO-*b*-PCCL/SN-38 in Figure 4A, between mPEO-*b*-PCCL/SN-38 and PEO-*b*-PBCL, between mPEO-*b*-PCCL/SN-38 and mPEO-*b*-PBCL/SN-38 in Figure 4B, and between free SN-38 and mPEO-*b*-PBCL/SN-38, between free SN-38 and mPEO-*b*-PCCL/SN-38 in Figure 4C. Significances of the differences were considered if *p* ≤ 0.05. Data are expressed as mean ± SD (*n* = 3).

Using DLS, the kinetic stability of block copolymeric micelles was investigated in the presence of a destabilizing surfactant, i.e., SDS, over 24 h. Figure 4A,B represent the percentage of intensity of the micellar peak and PDI, respectively, for mPEO-*b*-PBCL, mPEO-*b*-PCCL, mPEO-*b*-PBCL/SN-38, and mPEO-*b*-PCCL/SN-38 micelles over time (1, 2, 4, 8, and 24 h) in the presence of SDS. Notably, mPEO-*b*-PBCL and mPEO-*b*-PBCL/SN-38 micelles exhibited complete resistance against the destabilizing agent and remained intact throughout the incubation period up to 24 h. In contrast, mPEO-*b*-PCCL micelles were completely dissociated right after mixing with SDS and the detected intensity of the micellar peak was below 1%. Due to complete dissociation, no PDI data were detectable by DLS at this time point, as shown in Figure 4B. In the case of mPEO-*b*-PCCL/SN-38, a dramatic drop in the percentage intensity of the micellar peak was observed immediately after SDS incorporation and 82% dissociation was recorded within 8 h. After 24 h, 0% intensity of micellar peak was recorded due to 100% dissociation of mPEO-*b*-PBCL/SN-38 micelles in the presence of SDS.

As shown in Figure 4B, both mPEO-*b*-PBCL and mPEO-*b*-PBCL/SN-38 micelles showed no change in PDI values obtained by DLS throughout the entire incubation period with SDS. In the case of mPEO-*b*-PCCL/SN-38 micelles, PDI values gradually increased above 0.8 within 8 h and reached to 1 after 24, implying complete micellar dissociation in the presence of the destabilizing agent. Overall, the data suggest that mPEO-*b*-PBCL-based micellar formulation is kinetically more stable than their PEO-*b*-PCCL counterparts.

### 3.4. In Vitro Drug Release

Figure 4C and Supplementary Materials Figure S4 show the comparative in vitro release profile of SN-38 from mPEO-*b*-PBCL/SN-38 and mPEO-*b*-PCCL/SN-38 micelles versus free SN-38. Within 6 h, 92.3% drug was rapidly released from the dialysis bag containing free SN-38 solubilized using DMSO. Such an apparent burst release indicates a condition where diffusion controls the release of the drug [30]. In contrast, only 26.8% and 33.8% of SN-38 were released over 6 h from mPEO-*b*-PBCL/SN-38 and mPEO-*b*-PCCL/SN-38 micelles, respectively. In addition, the release of SN-38 from mPEO-*b*-PBCL/SN-38 micelles over 24 h was 36.5%, which was significantly lower than that of mPEO-*b*-PCCL/SN-38 micelles (47%) (Unpaired Student’s *t*-test, * *p* < 0.05). After 24 h, the release of free SN-38 was 96%. At 48 and 72 h, the release SN 38 from both mPEO-*b*-PBCL/SN-38 and mPEO-*b*-PCCL/SN-38 continued to increase and reached around 55% and 65%, on average, respectively (Figure S4). The difference between the SN-38 release from the two formulations at 48 and 72 h was not statistically significant (*p* > 0.05).

The 24 h release profiles between free SN-38, mPEO-*b*-PBCL/SN-38, and mPEO-*b*-PCCL/SN-38 micelles were analyzed by measuring the difference (f_1_) and similarity (f_2_) factors (Table 2). As a result, calculated f_1_ and f_2_ values were found above 15 and below 50, respectively, when the percent release of free SN-38 was compared with that of both micellar SN-38 formulations. However, the calculated f_2_ values were found to be above 50 when the release profiles between mPEO-*b*-PBCL/SN-38 and mPEO-*b*-PCCL/SN-38 micelles were compared, suggesting similar release kinetics. The overall in vitro release study supports the efficiency of micellar formulations for sustained release of SN-38.

### 3.5. In Vitro Cytotoxicity

**Figure 5:**
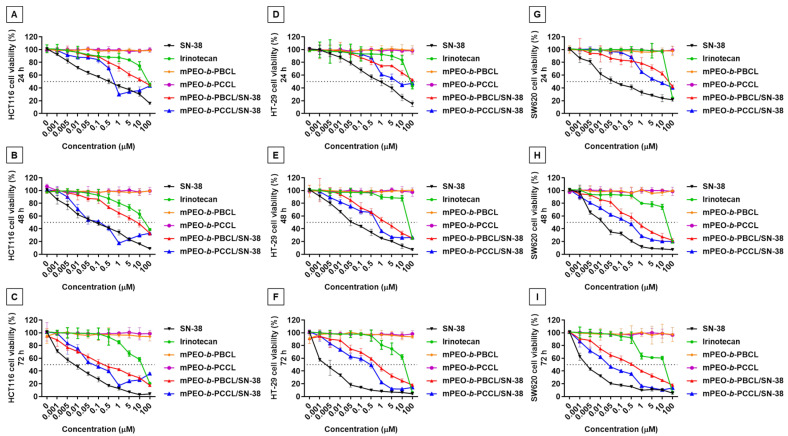
In vitro cytotoxicity assay for free SN-38 (black), irinotecan (green), mPEO-*b*-PBCL (orange), mPEO-*b*-PCCL (purple), mPEO-*b*-PBCL/SN-38 (red), and mPEO-*b*-PCCL/SN-38 (blue) in (**A**–**C**) HCT116, (**D**–**F**) HT-29, and (**G**–**I**) SW620 cell lines after 24 h, 48 h, and 72 h incubation at 37 °C in 5% CO_2_. The cells were treated with free drug and polymeric micelles with a range of concentrations from 0.001 µM to 100 µM. SN-38 was solubilized with DMSO and the untreated cells received only 0.1% DMSO. Each point represents mean ± SD (*n* = 4).

The MTS assay was performed up to 72 h to determine the anti-cancer activity of micellar mPEO-*b*-PBCL/SN-38 and mPEO-*b*-PCCL/SN-38 formulations against CRC cell lines (HCT116, HT-29, SW620) in comparison to empty polymeric micelles, free SN-38, and irinotecan. Figure 5 shows the average percentages of cell survival over 24, 48, and 72 h treatment with the formulations. The ranges for IC_50_s for each of the treatments at different incubation time points are summarized in Table 3. As shown in Figure 5, free SN-38, irinotecan, mPEO-*b*-PBCL/SN-38, and mPEO-*b*-PCCL/SN-38 showed time and dose-dependent cytotoxicity against all cell lines under study. However, no toxicity was observed for drug-free mPEO-*b*-PBCL and mPEO-*b*-PCCL. SN-38 solubilized with DMSO exhibited the highest toxicity against all cell lines. The HCT116 CRC cell line showed the least sensitivity to free SN-38 (IC_50_: 0.01 ± 0.002 µM) over 72 h incubation when compared to that of HT-29 (IC_50_: 0.002 ± 0.001 µM) and SW620 (IC_50_: 0.002 ± 0.001 µM) cell lines. Both mPEO-*b*-PBCL/SN-38 and mPEO-*b*-PCCL/SN-38 treatments exhibited a significant reduction in the viability of CRC cell lines under study over 72 h when compared to irinotecan. The calculated IC_50_ values for irinotecan were 6.94 ± 2.51, 11.35 ± 4.04, and 6.63 ± 3.64 µM after 72 h incubation with HCT116, HT-29, and SW620 cell lines, respectively. The obtained IC_50_ values for mPEO-*b*-PBCL/SN-38 micellar treatments were 0.11 ± 0.04 µM (HCT116), 0.39 ± 0.16 µM (HT-29), and 0.10 ± 0.04 µM (SW620) for 72 h of treatment. For mPEO-*b*-PCCL/SN-38 after a similar treatment period, the calculated IC_50_s were 0.04 ± 0.02 µM (HCT116), 0.08 ± 0.04 µM (HT-29), and 0.02 ± 0.01 µM (SW620). Overall, the CRC cells under study were, on average, 70-fold to 330-fold more sensitive to the polymeric micellar conjugates of SN-38 developed here than irinotecan. There was no significant difference between the IC_50_ of mPEO-*b*-PCCL/SN-38 and mPEO-*b*-PBCL/SN-38 micelles over 72 h in all three CRC cell lines. The similar trend in cell viability reduction observed for the SN-38-conjugated micellar formulations; suggests that both mPEO-*b*-PBCL/SN-38 and mPEO-*b*-PCCL/SN-38 micelles effectively preserved and delivered the active structure of SN-38 to the CRC cell lines.

### 3.6. Caspase 3/7 Activity

**Figure 6:**
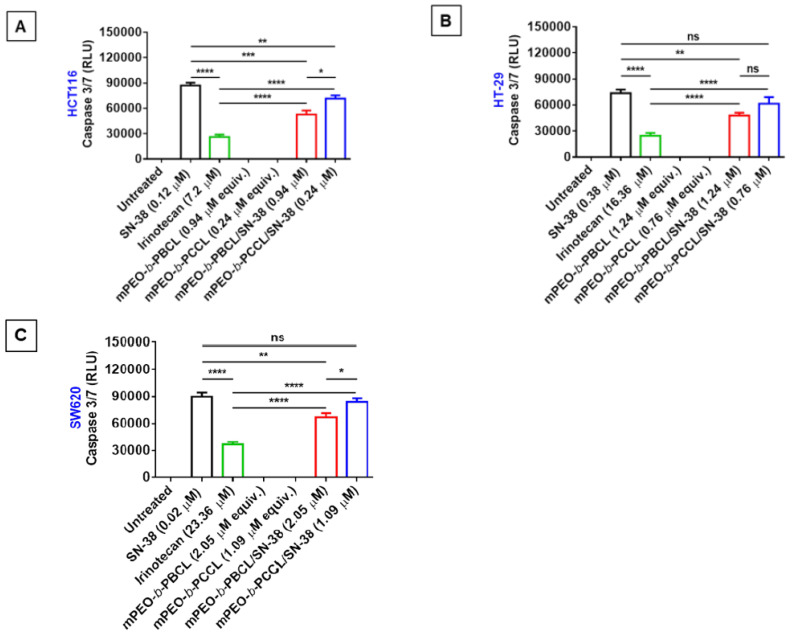
Caspase activity assay for free SN-38, irinotecan, mPEO-*b*-PBCL, mPEO-*b*-PCCL, mPEO-*b*-PBCL/SN-38, and mPEO-*b*-PCCL/SN-38 in (**A**) HCT116, (**B**) HT-29, and (**C**) SW620 cell lines. The cells were treated with the media containing the respective IC_50_ (24 h) concentrations of free SN-38, irinotecan, mPEO-*b*-PBCL/SN-38, and mPEO-*b*-PCCL/SN-38 for 6 h. Treated amounts of mPEO-*b*-PBCL and mPEO-*b*-PCCL were equivalent to the amounts of mPEO-*b*-PBCL/SN-38 and mPEO-*b*-PCCL/SN-38, respectively. The untreated (control) cells received only 0.1% DMSO. The significances of the differences are indicated as * *p* ≤ 0.05, ** *p* ≤ 0.01, *** *p* ≤ 0.001, and **** *p* ≤ 0.0001 following a two-way ANOVA multiple comparison test, which was followed by Tukey’s method. Data are expressed as mean ± SD (*n* = 6). ns: no significance.

As shown in Figure 6, significantly higher caspase-3/7 activation was observed with free SN-38, irinotecan, mPEO-*b*-PBCL/SN-38, and mPEO-*b*-PCCL /SN-38 treatments at their respective IC_50_s (24 h) when compared to untreated or drug-free mPEO-*b*-PBCL and mPEO-*b*-PCCL-treated cells. At their respective IC_50_s, mPEO-*b*-PBCL/SN-38 and mPEO-*b*-PCCL/SN-38 micellar treatments resulted in ≈two-fold higher activation of caspase-3/7 when compared to irinotecan. Notably, no statistically significant difference was observed between caspase 3/7 activation by free SN-38 and mPEO-*b*-PCCL/SN-38 in HT-29 and SW620 cell lines. The mPEO-*b*-PBCL/SN-38 formulation, however, showed less activity in this regard when compared to free SN-38 in all cell lines under study. A small, but significant, difference between the caspase 3/7 activation of the two micellar SN-38 formulations was observed in the HCT116 and SW620 cell lines, but the difference in the HT-29 cell line did not reach significance. Overall, results strongly demonstrate that, unlike irinotecan, the free SN-38, mPEO-*b*-PBCL/SN-38, and mPEO-*b*-PCCL/SN-38 micelles trigger the enzymatic caspase-3/7 activation pathway that promotes the cellular apoptosis in CRC cell lines.

### 3.7. Hemolytic Activity Assessment

**Figure 7:**
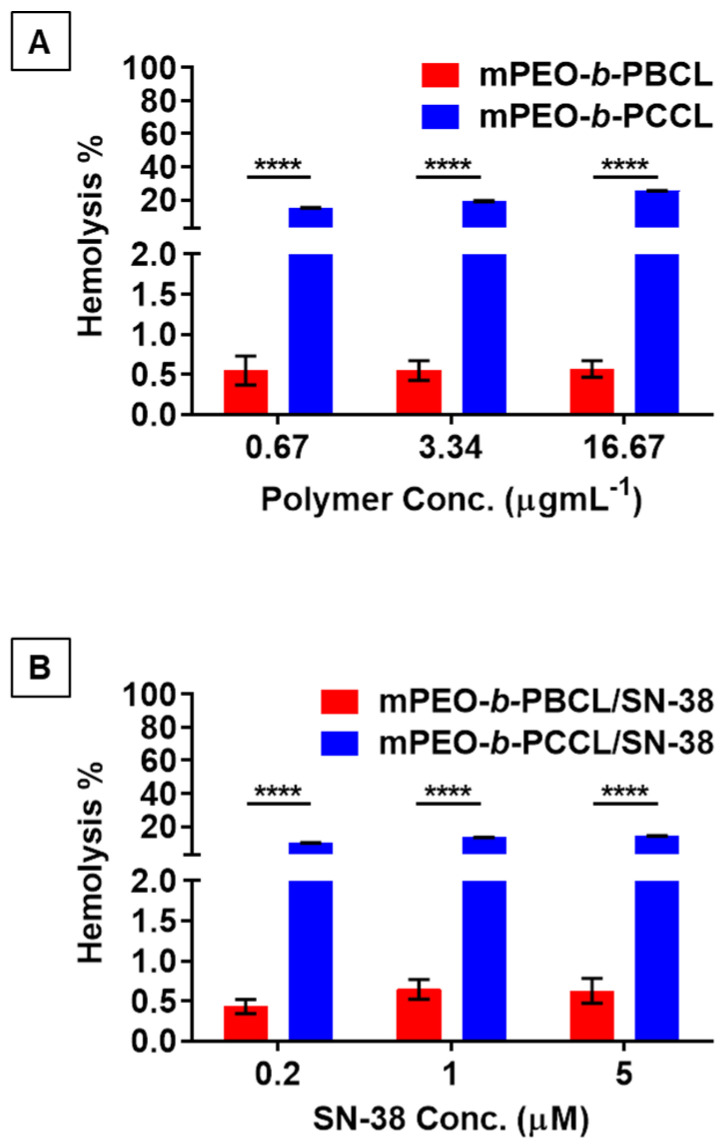
Hemolytic activity of (**A**) mPEO-*b*-PBCL and mPEO-*b*-PCCL, (**B**) mPEO-*b*-PBCL/SN-38 and mPEO-*b*-PCCL/SN-38 micellar formulations against rat red blood cells (RBCs). Each error bar represents the mean ± SD (*n* = 3). The concentrations of the polymers in the micellar formulations (0.67, 3.34, and 16.67 µg·mL^−1^) were equivalent to their respective concentration in the SN-38-conjugated formulations. SN-38 concentration of 0.2 (5 times less than IC_50_), 1 (≈IC_50_), and 5 µM (five times higher than IC_50_) and their equivalent polymer concentrations were chosen for the study. Isotonic PBS and full hemolysis by pure water were used as negative and positive controls, respectively. All marked points were compared and were statistically significant if **** *p* ≤ 0.0001.

As shown in Figure 7A, 30 min exposure to mPEO-*b*-PBCL did not exhibit any hemolytic activity at a polymer concentration as high as 16.67 µg·mL^−1^, which was the polymer concentration equivalent to the highest concentration of SN-38 (five times higher than its IC_50_). Similarly in Figure 7B, mPEO-*b*-PBCL/SN-38 showed no hemolytic activity at a concentration of 5 µM that was five times higher than its IC_50_ (1 µM after 24 h of treatment). However, mPEO-*b*-PCCL showed 15.32 ± 0.18%, 19.42 ± 0.34%, and 25.63 ± 0.13% hemolysis at 0.67 µg·mL^−1^, 3.34 µg·mL^−1^, and 16.67 µg·mL^−1^ concentrations, respectively, which was significantly (*p* ≤ 0.0001) higher than the hemolytic activities caused by mPEO-*b*-PBCL at similar levels (Figure 7A). The measured percentages of hemolytic activity of mPEO-*b*-PCCL/SN-38 micelles were 10.19 ± 0.15, 13.45 ± 0.14, and 14.46 ± 0.15 at polymer concentrations of 0.67 µg·mL^−1^, 3.34 µg·mL^−1^, and 16.67 µg·mL^−1^, respectively, which was significantly (*p* ≤ 0.05) reduced when compared to that of mPEO-*b*-PCCL micelles after SN-38 conjugation (Figure 7B). The overall results suggest that mPEO-*b*-PBCL and its SN-38 conjugated form are unlikely to affect red blood cells [31]. In contrast, mPEO-*b*-PCCL and its SN-38 conjugated form can cause hemolysis despite a reduction in the hemolytic activity of the polymer upon SN-38 conjugation.

## 4. Discussion

Irinotecan is a clinically used chemotherapeutic agent in CRC patients but it causes dose-dependent unwanted toxicities. The adverse side-effects of irinotecan are partly due to high required doses of the drug necessitated by irinotecan’s inefficient (<10%) conversion to its active form (SN-38) in liver and cancer cells. The main objective of this study was to develop a micellar delivery system for the active metabolite of irinotecan, SN-38, so that its solubility in water can be improved and its delivery to cancerous cells can be enhanced. To this end, SN-38 was chemically conjugated to the poly(ester) core of two different micellar formulations, i.e., mPEO-*b*-PBCL and mPEO-*b*-PCCL (Figure 1). The effect of the chemistry of the poly(ester) core in these SN-38 polymeric micellar-drug conjugates on the physicochemical and functional characteristics of these nano-delivery systems of SN-38 was then investigated, in vitro, in order to select an optimum formulation for future in vivo studies.

The water solubility of SN-38 is around 25 μg·mL^−1^. The achieved solubilized levels by the SN-38 polymer conjugates were >10 mg·mL^−1^, which is >400-fold higher than SN-38 water solubility and meets the criteria for systemic therapy [32]. This was through the formation of associated colloidal delivery systems of SN-38 with sufficient thermodynamic and kinetic stability in an aqueous medium. Moreover, chemical conjugation or physical encapsulation of SN-38 into various delivery systems is challenging due to the inherent poor solubility of SN-38 in organic solvents mostly used for the chemical reaction and solubilization depending on the types of delivery formulation [33,34,35,36]. This limitation was shown to impede high loading and loading efficiency of SN-38 into the delivery systems. Notably, only 20% (*w/w*) SN-38 loading was observed in NK012 formulation, which is the only SN-38-conjugated PEG-poly(glutamic acid) micellar nano-carrier under clinical trial phase-II investigation [21,37]. The conjugation level of SN-38 in mPEO-*b*-PBCL/SN-38 and mPEO-*b*-PCCL/SN-38 under this study were <15% (*w/w*), pointing to the necessity for further optimization to increase the loading despite the high potency of SN-38. Higher drug conjugation levels were expected for PEO-*b*-PCCL when compared to the PEO-*b*-PBCL due to the existence of higher pendent functional COOH groups in PCCL-based polymer compared to that for the PBCL polymer, which only holds one COOH group at the end of the polymer chain. In practice, we observed a slightly higher SN-38 loading in PCCL-based polymer than PBCL-based polymer. However, no significant difference was observed after statistical analysis.

In previous studies, a proportional correlation was identified between the hydrophobicity of block copolymers and the kinetic stability of polymeric micelles [38,39]. An increase in the hydrophobicity of the copolymers, either by core-forming block elongation (higher degree of polymerization) or by introducing more hydrophobic pendant or end groups, was shown to reduce the CMC, aggregation, dissociation rate, and hydrolytic degradation of formed micelles as a result of improved thermodynamic and kinetic stability. In line with previous observations, conjugation of hydrophobic SN-38 to the core of both polymeric micellar formulations under study here led to improved thermodynamic and kinetic stability of micelles. However, mPEO-b-PBCL/SN-38 micelles showed significantly lower CMC and superior kinetic stability than that of mPEO-b-PCCL/SN-38. This could be due to the presence of aromatic and more hydrophobic benzyl pendant groups in the mPEO-*b*-PBCL and intra-micellar benzyl π-π stacking interactions when compared to the pendant carboxyl group in the mPEO-b-PCCL/SN-38 structure [24,38]. In agreement with the above observation, the presence of the benzyl structure in the micellar core also contributed to the prevention of micellar dissociation by destabilizing agents by making the core more rigid. In contrast, more rapid dissociation and PDI elevation of mPEO-*b*-PCCL/SN-38 micelles have been observed, owing to the presence of hydrophilic carboxyl pendent groups in the core structure. In addition, the COOH groups of the PCCL backbone, being partially ionized at neutral pH, could be responsible for micellar swelling and dissociation due to the generation of repulsive forces between similarly charged -COOH groups [40,41].

In our study, the release of SN-38 from mPEO-*b*-PBCL/SN-38 and mPEO-*b*-PCCL/SN-38 micelles was significantly slower than free SN-38 from dialysis bags, validating the slow cleavage of the linkage between SN-38 and the polymeric backbone and/or cleavage of SN-38-caprolactone derivatives from the micellar core. Over 90% burst release of free SN-38 from the dialysis bag within 6 h demonstrated that the release of the drug from the dialysis bag to the release medium was not a rate-limiting factor. We used a 3500 Da MW cut off for the dialysis membrane that does not allow passage of an SN-38 conjugated polymer or micelles to the recipient media, but cleaved SN-38 and/or SN-38-attached to caprolactone oligomers and/or derivatives, resulting from the degradation of the polymeric backbone, can easily pass through the dialysis membrane. Despite the lower hydrophobicity of the PCCL compared to the PBCL core, the release profiles of SN-38 from both micellar structures were similar. This may reflect similar kinetics of the SN-38-polymer link cleavage within the micellar core since the release study was performed above the CMC of each polymeric-drug conjugate.

We then investigated the cytotoxicity of polymer-SN-38 micellar conjugates to assess whether the released SN-38 species from the formulations and/or the polymer-drug conjugates in the intact form are bioactive and have equal potency to that of free SN-38. The cytotoxicity resulting from SN-38-incorporated micelles followed a similar trend as observed in the release study. Both SN-38-conjugated micellar formulations resulted in a similar viability reduction in human CRC cell lines (Figure 5). The free SN-38 was more cytotoxic than micellar SN-38. This was expected due to the sustained release of SN-38 from mPEO-*b*-PBCL/SN-38 and mPEO-*b*-PCCL/SN-38 micelles. Furthermore, both micellar SN-38 formulations were shown to be more cytotoxic than irinotecan in human CRC cell lines due to a more efficient release of active SN-38.

To confirm the in vitro mechanism of cytotoxic action for micellar SN-38 formulations, their ability to induce caspase 3/7 was tested in comparison to free SN-38 or irinotecan. The study was conducted at respective IC_50_ concentrations (24 h) of these drugs, so that a comparison of the mode of cytotoxic drug action rather than drug potency could be made. It is evident that caspases, which are a family of conserved proteases, play an important role in programmed cell death [42]. Polymeric micellar SN-38 significantly enhanced the caspase-3/7 activation levels when compared to irinotecan in all the CRC cell lines at their respective IC_50_s. A higher caspase-3/7 activation level for mPEO-*b*-PCCL/SN-38 over mPEO-*b*-PBCL/SN-38 treatment in some CRC cells could be a reflection of higher micellar kinetic stability of mPEO-*b*-PBCL/SN-38, which may eventually lead to lower drug release/core cleavage, extracellularly or intracellularly when compared to that for the PCCL/SN-38 based formulation [23]. The difference in cytotoxicity and caspase-3/7 activation of the two formulations may also be due to differences in the intracellular trafficking of the polymer/SN-38 conjugates or SN-38 released species from the two formulations. Further studies are required to elucidate this point.

In general, drug delivery systems in systemic circulation are expected to be in contact with RBCs, which is the most abundant cellular constituent of the blood [43,44]. From a safety viewpoint, it is indispensable to investigate the effects of delivery systems on cells encountered throughout the pathway to reach the site of therapeutic targets prior to preclinical and clinical studies. Therefore, SN-38 micellar formulations were tested for hemolytic activity. In our study, mPEO-*b*-PBCL/SN-38 micelles showed no hemolytic activity. On the other hand, mPEO-*b*-PCCL/SN-38 micelles resulted in relatively higher percentages of hemolysis at various concentrations under study. As previously investigated, the hemolytic activity (<15% at IC_50_ concentration) of mPEO-*b*-PCCL/SN-38 could be attributed to the presence of ionized pendent -COOH groups [45]. This was evident as the hemolytic activity of drug-free mPEO-*b*-PCCL micelles reached >25%, supporting the role of –COOH functional group-associated hemolytic activity [46].

## 5. Conclusions

In summary, in this study, the chemical conjugation of SN-38 to the hydrophobic core of self-associating PEO-poly(ester) micelles through two different strategies was successfully performed. This led to aqueous solubilized levels of SN-38 that are suitable for systemic administration in the form of PEO-poly(ester)-based nanocarriers in vivo animal models of CRC. Between the two polymeric micellar formulations, those with pendent SN-38/and free COOH groups, i.e., mPEO-*b*-PCCL/SN-38 were found to be less kinetically and thermodynamically stable and hemolytic. Both nano-carriers maintain the cytotoxic activity and mechanism of action of SN-38 in CRC cells in vitro. The overall findings point to the superiority of mPEO-*b*-PBCL/SN-38 micellar formulation as a potential delivery system for SN-38 against CRC.

## Figures and Tables

**Table 1:** Physicochemical characteristics of the self-assembled block copolymers and SN-38-conjugated block copolymer micelles (*n* = 4).

Micellar Formulations ^a^	Size ^b^ ± SD (nm)	PDI ^c^ ± SD	Zeta Potential ^d^ ± SD (mV)	CMC ^e^ ± SD (µg·mL^−1^)	SN-38 Loading ^f^ (% *w/w*)
mPEO_114_-*b*-PBCL_12_	46.25 ± 0.11	0.12 ± 0.01	0.09 ± 0.03	4.43 ± 0.21	-
mPEO_114_-*b*-PBCL_12_/SN-38	43.60 ± 0.14 ^g^	0.13 ± 0.01	−1.14 ± 0.23 ^g^	3.88 ± 0.11 ^g^	11.47 ± 0.10
mPEO_114_-*b*-PCCL_20_	56.76 ± 0.41	0.17 ± 0.01	0.04 ± 0.01	69.92 ± 0.82	-
mPEO_114_-*b*-PCCL_20_/SN-38	38.47 ± 0.34 ^g^	0.11 ± 0.02	−1.69 ± 0.18 ^g^	54.57 ± 0.12 ^g^	12.03 ± 0.17

^a^ The number shown in the subscript indicates the degree of polymerization of each block as determined by ^1^H NMR spectroscopy. ^b^ Hydrodynamic diameter (Z average) determined by dynamic light scattering (DLS). ^c^ Average polydispersity index (PDI) of micellar size distribution. ^d^ Average surface charge (zeta potential) of the micelles. ^e^ Average critical micellar concentration (CMC) measured by DLS. ^f^ SN-38 $\mathrm{loading}\;\left(w/w\;\%\right)=\frac{\mathrm{Amount}\;\mathrm{of}\;\mathrm{conjugated}\;\mathrm{SN}-38}{\mathrm{Total}\;\mathrm{amount}\;\mathrm{of}\;\mathrm{polymer}}\times 100$; measured using UV–Vis spectrophotoscopy. ^g^ Differences were significant when compared to their counterpart polymeric micelles without SN-38.

**Table 2:** Calculated difference factor (f_1_) and similarity factor (f_2_) for SN-38 release profiles from mPEO-*b*-PBCL/SN-38 and mPEO-*b*-PCCL/SN-38 micellar formulations. The profiles were considered similarly if f_1_ ≤ 15 and f_2_ ≥ 50.

Formulations	Difference Factor (f_1_)	Similarity Factor (f_2_)
Free SN-38 and mPEO_114_-*b*-PBCL_12_	75.01	8.73
Free SN-38 and mPEO_114_-*b*-PCCL_20_/SN-38	68.27	10.65
mPEO_114_-*b*-PBCL_12_/SN-38 and mPEO_114_-*b*-PCCL_20_/SN-38	26.95	56.97

**Table 3:** IC_50_ range of free SN-38, irinotecan, mPEO-*b*-PBCL/SN-38, and mPEO-*b*-PCCL/SN-38 against HCT116, HT-29, and SW620 cell lines after 24, 48, and 72 h of incubation (*n* = 4). IC_50_ values were determined after plotting the cell viability percentages vs. drug concentrations using GraphPad Prism 6 software. The graph was then fitted with a non-linear regression and sigmoid dose-response curve to obtain the IC_50_ values.

Cells	Time (h)	SN-38 (µM)	Irinotecan (µM)	mPEO-*b*-PBCL/SN-38 (µM)	mPEO-*b*-PCCL/SN-38 (µM)
HCT116	24	0.092–0.145	4.135–12.550	0.696–1.262	0.189–0.425
	48	0.039–0.065	3.367–6.409	0.711–1.308	0.032–0.067
	72	0.007–0.009	5.387–8.941	0.084–0.148	0.027–0.059
HT-29	24	0.296–0.496	8.622–31.060	0.742–2.069	0.601–0.963
	48	0.038–0.056	14.120–27.760	0.337–0.662	0.0986–0.195
	72	0.001–0.003	8.847–14.560	0.294–0.526	0.062–0.115
SW620	24	0.016–0.028	10.470–52.080	1.039–4.041	0.920–1.300
	48	0.012–0.018	8.532–18.220	0.171–0.303	0.038–0.059
	72	0.002–0.003	4.537–9.680	0.0792–0.136	0.018–0.028

## Supplementary Materials

The following are available online at https://www.mdpi.com/1999-4923/12/11/1033/s1, Figure S1. ^1^H NMR spectra of SN-38, Figure S2. ^1^H NMR spectra of mPEO-*b*-PBCL, Figure S3. ^1^H NMR spectra of mPEO-*b*-PCCL, Figure S4. The drug release profile of mPEO-*b*-PBCL/SN-38 and mPEO-*b*-PCCL/SN-38 micelles compared to free SN-38 from dialysis tubing (MWCO = 3.5 kDa) in 4% albumin in ultrapure water at 37 °C after 48 and 72 h.

## Abbreviations

BCL	α-benzyl carboxylate-ε-caprolactone
BSA	bovine serum albumin
CDCl3	deuterated chloroform
CMC	critical micellar concentration
CRC	colorectal cancer
DIC	N,N′-diisopropylcarbodiimide
DLS	dynamic light scattering
DMAP	4-dimethylaminopyridine
DMEM/F12	dulbecco’s modified eagle medium and F12
DMF	dimethylformamide
DMSO	dimethyl sulfoxide
DNA	deoxy ribonucleic acid
DP	degree of polymerization
EPR	enhanced permeability and retention effect
Kcps	kilo counts per second
mPEO	methoxy-polyethylene oxide
mPEO-*b*-PBCL	methoxy-poly(ethylene oxide)-b-poly(α-benzyl carboxylate-ε-caprolactone)
mPEO-*b*-PBCL/SN-38	SN-38-incorporated mPEO-b-PBCL micelle
mPEO-*b*-PCCL	methoxy-poly(ethylene oxide)-b-poly(α-carboxyl-ε-caprolactone)
mPEO-*b*-PCCL/SN-38	SN-38-incorporated mPEO-b-PCCL micelle
MW	molecular weight
PDI	polydispersity index
RBC	red blood cell
SDS	sodium dodecyl sulfate
SN-38	7-ethyl-10-hydroxy-camptothecin
TEM	transmission electron microscopy
THF	tetrahydrofuran
Topo-I	topoisomerase I
ZP	zeta potential

## References

[1] Kuipers E.J., Rösch T., Bretthauer M. (2013). Colorectal cancer screening—Optimizing current strategies and new directions. Nat. Rev. Clin. Oncol..

[2] Bray F., Me J.F., Soerjomataram I., Siegel R.L., Torre L.A., Jemal A. (2018). Global cancer statistics 2018: GLOBOCAN estimates of incidence and mortality worldwide for 36 cancers in 185 countries. CA Cancer J. Clin..

[3] Kuipers E.J., Grady W.M., Lieberman D., Seufferlein T., Sung J.J., Boelens P.G., van de Velde C.J.H., Watanabe T. (2015). Colorectal cancer. Nat. Rev. Dis. Primers.

[4] Li X.-X., Zheng H.-T., Peng J.-J., Huang L.-Y., Shi D.-B., Liang L., Cai S.-J. (2014). RNA-seq reveals determinants for irinotecan sensitivity/resistance in colorectal cancer cell lines. Int. J. Clin. Exp. Pathol..

[5] Lee P.-C., Chiou Y.-C., Wong J.-M., Peng C.-L., Shieh M.-J. (2013). Targeting colorectal cancer cells with single-walled carbon nanotubes conjugated to anticancer agent SN-38 and EGFR antibody. Biomaterials.

[6] Inagaki Y., Yoshida Y., Hamasaki Y., Ueki H. (1991). Protooncogene (C-Myc) Expression in the Infiltrating Cells of Lesional Skin from Patients with Systemic Lupus Erythematosus. J. Investig. Dermatol..

[7] Roger E., Lagarce F., Benoit J.-P. (2011). Development and characterization of a novel lipid nanocapsule formulation of Sn38 for oral administration. Eur. J. Pharm. Biopharm..

[8] Gupta E., Lestingi T.M., Mick R., Ramirez J., Vokes E.E., Ratain M.J. (1994). Metabolic fate of irinotecan in humans: Correlation of glucuronidation with diarrhea. Cancer Res..

[9] Garcia-Carbonero R., Supko J.G. (2002). Current perspectives on the clinical experience, pharmacology, and continued development of the camptothecins. Clin. Cancer Res..

[10] Wang W., Ghandi A., Liebes L., Louie S.G., Hofman F.M., Schönthal A.H., Chen T.C. (2011). Effective conversion of irinotecan to SN-38 after intratumoral drug delivery to an intracranial murine glioma model in vivo. J. Neurosurg..

[11] Koizumi F., Kitagawa M., Negishi T., Onda T., Matsumoto S.-I., Hamaguchi T., Matsumura Y. (2006). Novel SN-38–Incorporating Polymeric Micelles, NK012, Eradicate Vascular Endothelial Growth Factor–Secreting Bulky Tumors. Cancer Res..

[12] Matsumura Y., Kataoka K. (2009). Preclinical and clinical studies of anticancer agent-incorporating polymer micelles. Cancer Sci..

[13] Dawidczyk C.M., Kim C., Park J.H., Russell L.M., Lee K.H., Pomper M.G., Searson P.C. (2014). State-of-the-art in design rules for drug delivery platforms: Lessons learned from FDA-approved nanomedicines. J. Control. Release.

[14] Estanqueiro M., Amaral M.H., Conceição J., Lobo J.M.S. (2015). Nanotechnological carriers for cancer chemotherapy: The state of the art. Colloids Surfaces B Biointerfaces.

[15] Pérez-Herrero E., Fernández-Medarde A. (2015). Advanced targeted therapies in cancer: Drug nanocarriers, the future of chemotherapy. Eur. J. Pharm. Biopharm..

[16] Kataoka K., Harada A., Nagasaki Y. (2001). Block copolymer micelles for drug delivery: Design, characterization and biological significance. Adv. Drug Deliv. Rev..

[17] Greenwald R.B., Pendri A., Conover C., Gilbert C., Yang A.R., Xia J. (1996). Drug Delivery Systems. 2. Camptothecin 20-O-Poly(ethylene glycol) Ester Transport Forms. J. Med. Chem..

[18] Wani M.C., Nicholas A.W., Manikumar G., Wall M.E. (1987). Plant antitumor agents. 25. Total synthesis and antileukemic activity of ring A substituted camptothecin analogues. Structure-activity correlations. J. Med. Chem..

[19] Pan X.-D., Liu H.-Y., Sun P.-Y., Zhu C.-G., Yang J., Yuan K.-H., Han R. (2004). Synthesis and antitumor activity of 20-O-linked camptothecin ester derivatives. Yao Xue Xue Bao Acta Pharm. Sin..

[20] Wang C.Y., Pan X.D., Liu H.Y., Wei X.Y., Yang L.X. (2004). Synthesis and antitumor activity of 20-O-linked nitrogen-based camptothecin ester derivatives. Bioorg. Med. Chem..

[21] Matsumura Y. (2011). Preclinical and clinical studies of NK012, an SN-38-incorporating polymeric micelles, which is designed based on EPR effect. Adv. Drug Deliv. Rev..

[22] Kenmotsu H., Yasunaga M., Goto K., Nagano T., Kuroda J.-I., Koga Y., Takahashi A., Nishiwaki Y., Matsumura Y. (2010). The antitumor activity of NK012, an SN-38-incorporating micelle, in combination with bevacizumab against lung cancer xenografts. Cancer.

[23] Mahmud A., Xiong X.-B., Lavasanifar A. (2008). Development of novel polymeric micellar drug conjugates and nano-containers with hydrolyzable core structure for doxorubicin delivery. Eur. J. Pharm. Biopharm..

[24] Garg S.M., Vakili M.R., Lavasanifar A. (2015). Polymeric micelles based on poly(ethylene oxide) and alpha-carbon substituted poly(varepsilon-caprolactone): An in vitro study on the effect of core forming block on polymeric micellar stability, biocompatibility, and immunogenicity. Colloids Surf. B Biointerfaces.

[25] Hakala R.A., Korhonen H., Holappa S., Seppälä J.V. (2009). Hydrophobicities of poly(ε-caprolactone) oligomers functionalized with different succinic anhydrides. Eur. Polym. J..

[26] Shire Z., Vakili M.R., Morgan T.D.R., Hall D.G., Lavasanifar A., Weinfeld M. (2018). Nanoencapsulation of Novel Inhibitors of PNKP for Selective Sensitization to Ionizing Radiation and Irinotecan and Induction of Synthetic Lethality. Mol. Pharm..

[27] Saqr A., Vakili M.R., Huang Y.-H., Lai R., Lavasanifar A. (2019). Development of Traceable Rituximab-Modified PEO-Polyester Micelles by Postinsertion of PEG-phospholipids for Targeting of B-cell Lymphoma. ACS Omega.

[28] Yu S., Huang Q., Luo Y., Lu W. (2011). Total Synthesis of Camptothecin and SN-38. J. Org. Chem..

[29] Cheng G., Liu Y., Piao H., Gao Y., Xu C., Tian Y., Wang L., Liu J., Tang B., Zou M. (2015). Comparison of two self-assembled macromolecular prodrug micelles with different conjugate positions of SN38 for enhancing antitumor activity. Int. J. Nanomed..

[30] Aliabadi H.M., Lavasanifar A. (2006). Polymeric micelles for drug delivery. Expert Opin. Drug Deliv..

[31] De La Harpe K.M., Kondiah P.P., Choonara Y.E., Marimuthu T., Du Toit L.C., Pillay V. (2019). The Hemocompatibility of Nanoparticles: A Review of Cell–Nanoparticle Interactions and Hemostasis. Cells.

[32] Kalepu S., Nekkanti V. (2015). Insoluble drug delivery strategies: Review of recent advances and business prospects. Acta Pharm. Sin. B.

[33] Goldberg D.S., Vijayalakshmi N., Swaan P.W., Ghandehari H. (2011). G3.5 PAMAM dendrimers enhance transepithelial transport of SN38 while minimizing gastrointestinal toxicity. J. Control. Release.

[34] Ebrahimnejad P., Dinarvand R., Jafari M.R., Tabasi S.A.S., Atyabi F. (2011). Characterization, blood profile and biodistribution properties of surface modified PLGA nanoparticles of SN-38. Int. J. Pharm..

[35] Ebrahimnejad P., Dinarvand R., Sajadi S.A., Atyabi F., Ramezani F., Jaafari M.R. (2010). Preparation and characterization of poly lactide-co-glycolide nanoparticles of SN-38. PDA J. Pharm. Sci. Technol..

[36] Ebrahimnejad P., Dinarvand R., Sajadi A., Jaafari M.R., Nomani A.R., Azizi E., Rad-Malekshahi M., Atyabi F. (2010). Preparation and in vitro evaluation of actively targetable nanoparticles for SN-38 delivery against HT-29 cell lines. Nanomedicine.

[37] Hamaguchi T., Tsuji A., Yamaguchi K., Takeda K., Uetake H., Esaki T., Amagai K., Sakai D., Baba H., Kimura M. (2018). A phase II study of NK012, a polymeric micelle formulation of SN-38, in unresectable, metastatic or recurrent colorectal cancer patients. Cancer Chemother. Pharmacol..

[38] Owen S.C., Chan D.P., Shoichet M.S. (2012). Polymeric micelle stability. Nano Today.

[39] Gaucher G., Dufresne M.-H., Sant V.P., Kang N., Maysinger D., Leroux J.-C. (2005). Block copolymer micelles: Preparation, characterization and application in drug delivery. J. Control. Release.

[40] Shen C., Guo S., Lu C. (2008). Degradation behaviors of monomethoxy poly(ethylene glycol)-b-poly(ɛ-caprolactone) nanoparticles in aqueous solution. Polym. Adv. Technol..

[41] Hu Y., Zhang L., Cao Y., Ge H., Jiang X., Yang C. (2004). Degradation behavior of poly(epsilon-caprolactone)-b-poly(ethylene glycol)-b-poly(epsilon-caprolactone) micelles in aqueous solution. Biomacromolecules.

[42] Boice A., Bouchier-Hayes L. (2020). Targeting apoptotic caspases in cancer. Biochim. Biophys. Acta Mol. Cell Res..

[43] Rossi L., Serafini S., Pierigè F., Antonelli A., Cerasi A., Fraternale A., Chiarantini L., Magnani M. (2005). Erythrocyte-based drug delivery. Expert Opin. Drug Deliv..

[44] Huang H., Lai W., Cui M., Liang L., Lin Y., Fang Q., Liu Y., Xie L. (2016). An Evaluation of Blood Compatibility of Silver Nanoparticles. Sci. Rep..

[45] Vo N.N.Q., Fukushima E.O., Muranaka T. (2016). Structure and hemolytic activity relationships of triterpenoid saponins and sapogenins. J. Nat. Med..

[46] Falamarzian A., Lavasanifar A. (2010). Chemical Modification of Hydrophobic Block in Poly(Ethylene Oxide) Poly(Caprolactone) Based Nanocarriers: Effect on the Solubilization and Hemolytic Activity of Amphotericin B. Macromol. Biosci..

