# Supplementary material for: Development of Self-Associating SN-38-Conjugated Poly(ethylene oxide)-Poly(ester) Micelles for Colorectal Cancer Therapy
Source: Pharmaceutics. 2020 Oct 29;12(11):1033. doi: 10.3390/pharmaceutics12111033 (PMC7694018; doi:10.3390/pharmaceutics12111033)
Supplement: Supplementary file 1 [file pharmaceutics-12-01033-s001.pdf]

pharmaceutics

MDPI

Supplementary Materials: Development of Self-Associating SN-38-Conjugated Poly(ethylene oxide)-poly(ester) Micelles for Colorectal Cancer Therapy

Sams M. A. Sadat, Mohammad Reza Vakili, Igor M. Paiva, Michael Weinfeld and Afsaneh Lavasanifar

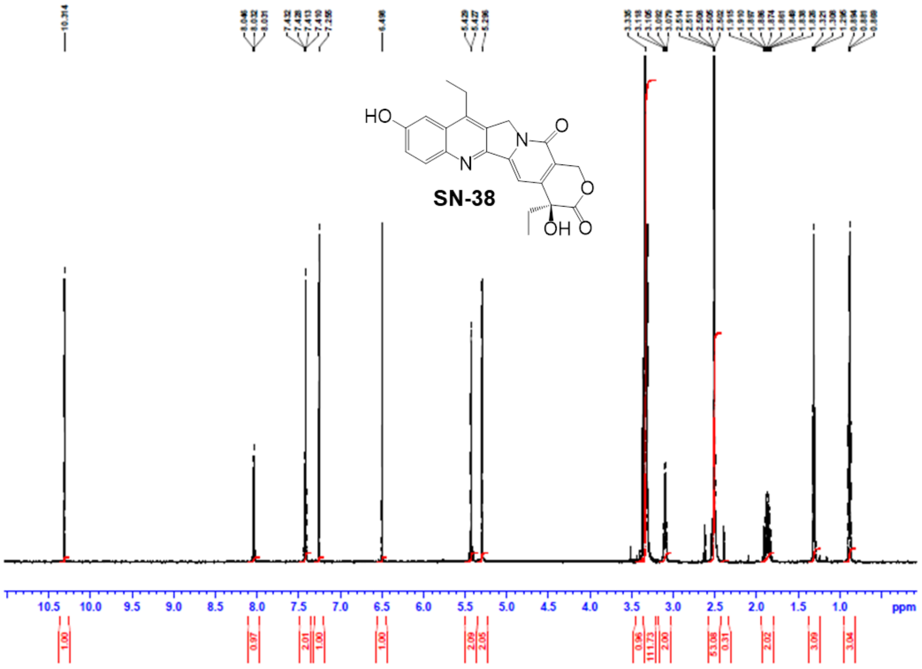

Figure S1. ^{1}H NMR spectra of SN-38.

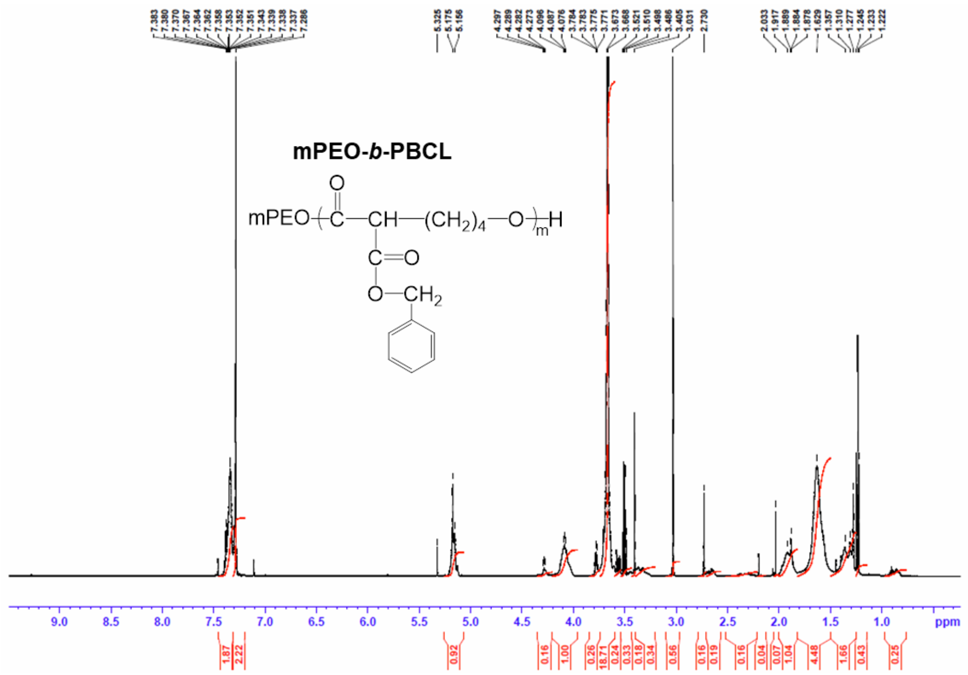

Figure S2. ^{1}H NMR spectra of mPEO-*b*-PBCL.

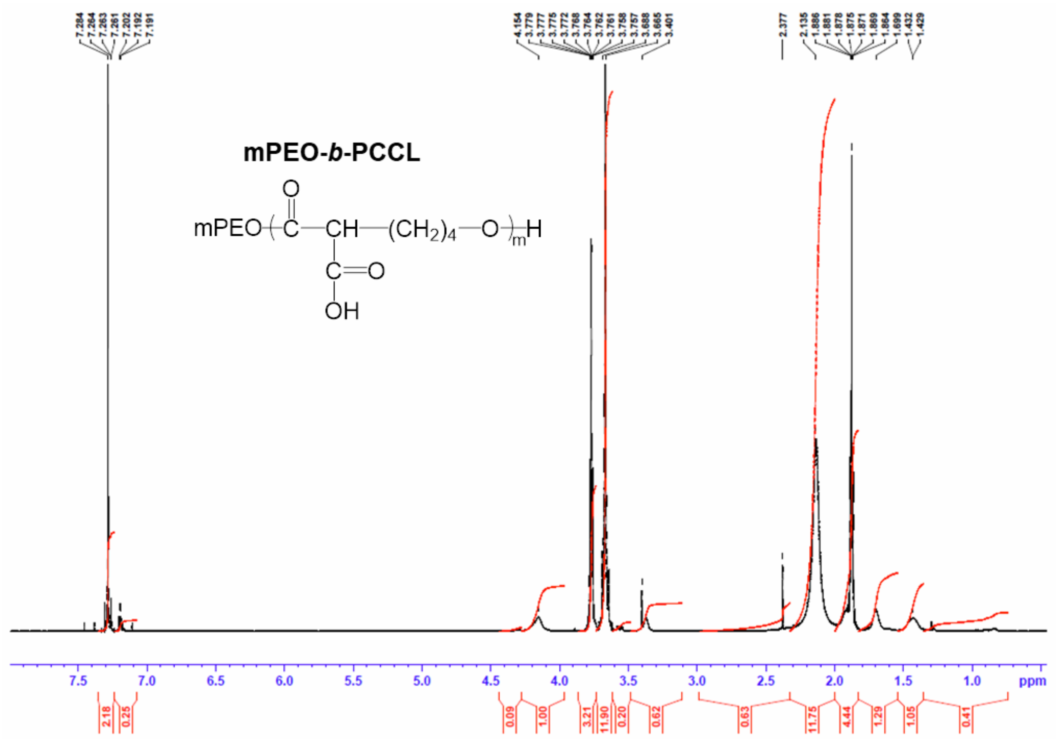

Figure S3. ^1H NMR spectra of mPEO-*b*-PCCL.

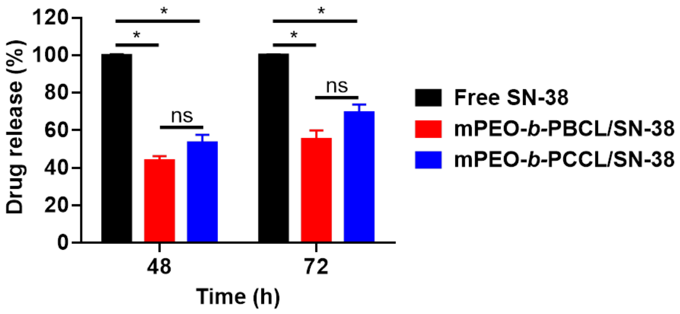

Figure S4. The drug release profile of mPEO-*b*-PBCL/SN-38 and mPEO-*b*-PCCL/SN-38 micelles compared to free SN-38 from dialysis tubing (MWCO = 3.5 kDa) in 4% albumin in ultrapure water at 37 °C after 48 and 72 h. Statistical analysis was operformed using two-way ANOVA followed by Tukey's post test. Data were considered significantly different if $p \leq 0.05$ and presented with *. Data are expressed as mean ± SD (n = 3).
